# Hypoxemia during veno-venous extracorporeal membrane oxygenation. When two is not better than one

**DOI:** 10.5935/0103-507X.v34n4-2022-ed-en

**Published:** 2022

**Authors:** António Tralhão, Philip Fortuna

**Affiliations:** 1 Cardiac Intensive Care Unit, Cardiology Department, Centro Hospitalar de Lisboa Ocidental, EPE - Carnaxide, Portugal.; 2 Medical Emergency Unit, Intensive Care Medicine Department, Centro Hospitalar de Lisboa Central, EPE - Lisboa, Portugal.

Unwittingly, hypoxemia may persist or even supervene after a patient is placed on veno-venous extracorporeal membrane lung oxygenation (VV-ECMO) for refractory hypoxemia. According to Extracorporeal Life Support Organization (ELSO) guidelines, the threshold for adequate arterial O_2_ saturation is > 80 - 85%,^(1)^ while a value > 88% has been considered the threshold in other guidelines.^(2)^ Although the exact incidence is difficult to ascertain and the definition itself may vary, hypoxemia during VV-ECMO requires both systematic assessment and prompt optimization of modifiable variables, as it has been associated with increased mortality.^(3)^ To fully understand why hypoxemia still occurs, one has to consider the principles underpinning the ability of ECMO to ensure adequate oxygen (O_2_) transfer across the membrane lung and into the patient’s blood. First, there is a fraction of oxygen in the fresh sweep gas that can be set, usually at 1.0. Second, a membrane lung, with an appropriate surface area available for gas exchange, needs to be working properly, allowing unimpeded blood flow around the gas-containing polymer microfibers. Third, the absolute amount of blood flowing through the oxygenator (Q_ECMO_) and its relative proportion to the patient’s own cardiac output (Q_patient_) need to be considered. Finally, the fraction of oxygenated blood flowing through ECMO that does not go into the pulmonary circulation but instead recirculates into the drainage cannula impacts the oxygenating efficacy of VV-ECMO.^(4)^

In a concept study, Schmidt et al. clearly demonstrated that blood flow through the ECMO circuit is the key determinant of blood oxygenation.^(5)^ Furthermore, as a higher proportion of deoxygenated venous blood goes through the patient’s right heart than through the ECMO circuit, the Q_ECMO_/Q_patient_ quotient falls below the boundary of 0.6, and the O_2_ content of arterial blood will drop even if the absolute blood flow through the membrane lung is appropriate to the body surface area.^(5)^ This is especially important if the degree of pulmonary shunt is such that any residual lung function contributing to oxygenation is negligible, which frequently occurs in patients being considered for VV-ECMO.^(4)^

To overcome persistent hypoxemia, different strategies have been devised. Among them, the most immediate would be to increase the Q_ECMO_/Q_patient_ ratio. Typical ECMO rated flows, which is the maximal flow at which hemoglobin [12g/dL] is fully saturated at the membrane outlet, are ~7L/minute. In these extreme situations, when a patient with no lung contribution and very high cardiac output has persistent severe hypoxemia or hypercarbia, adding a second oxygenator to the extracorporeal circuit, whether in parallel or in series, might be an intuitive option. In this issue of the *Revista Brasileira de Terapia Intensiva*, Melro et al.,^(6)^ using a porcine model, evaluated the impact on blood oxygenation of these two circuit configurations. Additionally, decarboxylation efficacy, as well as pressure and resistance changes to the circuit imposed by the “virtual” presence of a second oxygenator, were analyzed. To achieve this goal, the authors built on their own previous work^(7)^ by using a validated mathematical model to calculate peripheral arterial oxygen saturation, postoxygenator O_2_ content and arterial partial pressure of carbon dioxide (PaCO_2_) for different ECMO flows while keeping the remaining variables constant (pulmonary shunt fraction, ventilator fraction of inspired oxygen [FiO_2_], cardiac output, sweep gas flow, O_2_ fraction of sweep gas flow, hemoglobin concentration, O_2_ consumption and CO_2_ production).

The results were clear; whether in series or in parallel, a second oxygenator has little impact on the arterial O_2_ content, even with a rated ECMO flow as high as 6.5L/minute. For such a flow, postoxygenator partial pressure of oxygen is by definition ~500mmHg, limiting any relevant improvement in oxygenation, regardless of circuit configuration. In other words, more oxygenators do not mean more flow. For decarboxylation, because CO_2_ removal is mainly influenced by sweep gas flow, adding a second oxygenator decreased systemic CO_2_, an effect that was even more pronounced when an in-parallel configuration was used, since a higher inlet PaCO_2_ will also lead to improved CO_2_ clearance.^(8)^ Regarding pressures and resistances, the changes brought by a second oxygenator, whether in series or in parallel, are minimal when compared to a single oxygenator.

What are the implications of this study to clinical practice? Anecdotally, a few case reports have been published in which a second oxygenator was used in the setting of refractory hypoxemia during VV-ECMO, with inconsistent improvements in blood oxygenation.^(9,10)^ However, this was only achieved at the cost of unusually high ECMO flows (> 7L/min), mandating the placement of a second drainage cannula, with a consequent increase in both invasiveness and likelihood of access-related complications. However, and based on the results by Melro et al.,^(6)^ adding a second oxygenator should probably not be included in the possible strategies to improve oxygenation but instead might be used if adequate decarboxylation is not obtained with a high sweep gas flow and minimized CO_2_ production. Hence, when faced with hypoxemia during VV-ECMO, intensivists should consider other options. To aid in the decision process, some groups have proposed a stepwise approach.^(11,12)^ Considering the balance between oxygen delivery and consumption and the importance of Q_ECMO_/Q_patient_, these can range from hypothermia, neuromuscular blockade, prone positioning, packed red cell transfusion or beta-blockade to reduce cardiac output. Of these, prone positioning during VV-ECMO seems to be one of the most promising strategies, as it has been linked with not only improved oxygenation but also reduced mortality in observational studies. Ongoing randomized trials (NCT04139733, NCT04607551) may, in time, confirm whether the survival benefit from proning non-ECMO patients with ARDS will also apply to the ECMO population.

The study by Melro et al.^(6)^ has certain limitations, which have been duly acknowledged by the authors. Although their calculations are mathematically sound, they may not account for all the variables being considered, and importantly, only one type of oxygenator was used, followed by extrapolation to a two oxygenator model. Nevertheless, they are to be commended for answering a relevant clinical question that will not only steer us in the right direction but also likely contribute to better resource allocation in the future.
